# Mitochondrial genetic differentiation and morphological difference of *Miniopterus fuliginosus* and *Miniopterus magnater* in China and Vietnam

**DOI:** 10.1002/ece3.1428

**Published:** 2015-02-22

**Authors:** Shi Li, Keping Sun, Guanjun Lu, Aiqing Lin, Tinglei Jiang, Longru Jin, Joseph R Hoyt, Jiang Feng

**Affiliations:** 1 Jilin Provincial Key Laboratory of Animal Resource Conservation and Utilization, Northeast Normal UniversityChangchun, 130024, China; 2 College of Animal Science and Technology, Jilin Agricultural UniversityChangchun, 130118, China; 3 College of Urban and Environment Sciences, Changchun Normal UniversityChangchun, 130032, China; 4 Department of Ecology and Evolutionary Biology, University of CaliforniaSanta Cruz, California, 95064, USA

**Keywords:** *Miniopterus*, mitochondrial DNA, morphology, phylogeny, quaternary glaciation

## Abstract

Because of its complicated systematics, the bent-winged bat is one of the most frequently studied bat species groups. In China, two morphologically similar bent-winged bat species, *Miniopterus fuliginosus* and *Miniopterus magnater* were identified, but their distribution range and genetic differentiation are largely unexplored. In this study, we applied DNA bar codes and two other mitochondrial DNA genes including morphological parameters to determine the phylogeny, genetic differentiation, spatial distribution, and morphological difference of the *M. fuliginosus* and *M. magnater* sampled from China and one site in Vietnam. Mitochondrial DNA gene genealogies revealed two monophyletic lineages throughout the Tropic of Cancer. According to DNA bar code divergences, one is *M. fuliginosus* corresponding to the Chinese mainland and the other is *M. magnater* corresponding to tropical regions including Hainan and Guangdong provinces of China and Vietnam. Their most recent common ancestor was dated to the early stage of the Quaternary glacial period (ca. 2.26 million years ago [Ma] on the basis of D-loop data, and ca. 1.69–2.37 Ma according to ND2). A population expansion event was inferred for populations of *M. fuliginosus* at 0.14 Ma. The two species probably arose in separate Pleistocene refugia under different climate zones. They significantly differed in forearm length, maxillary third molar width, and greatest length of the skull.

## Introduction

**Figure 1:**
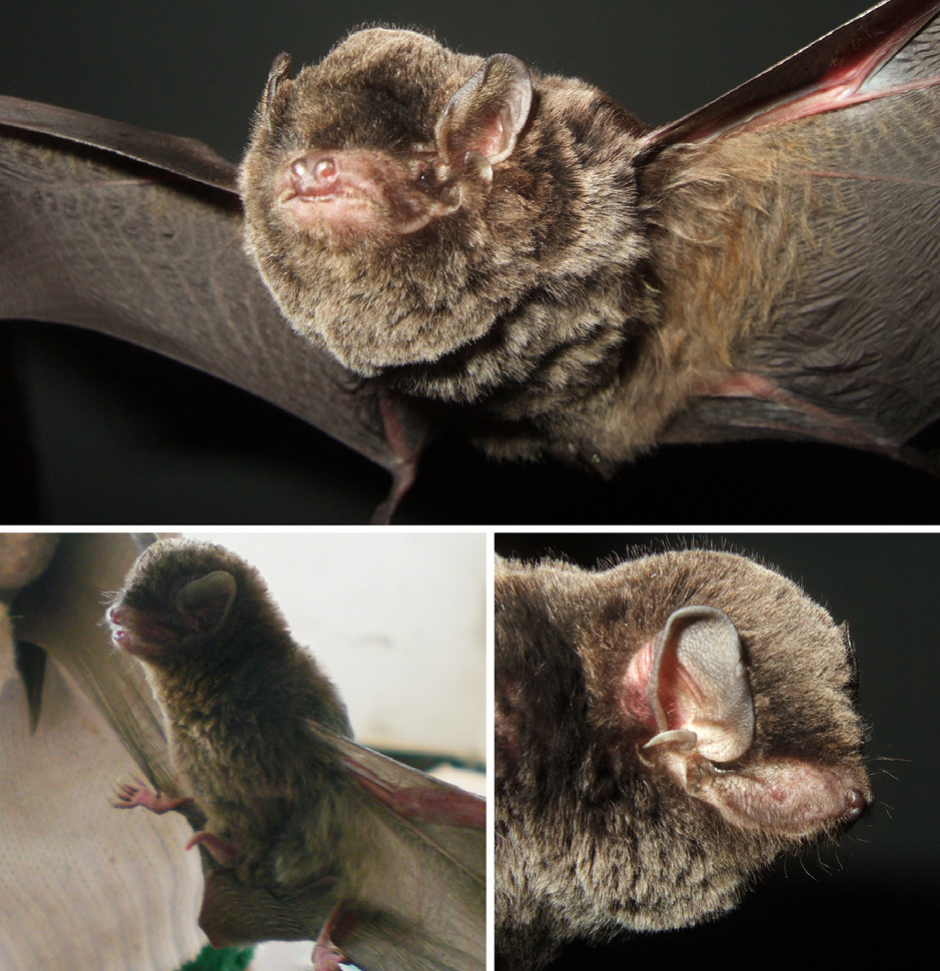
The bent-winged bat species, *Miniopterus fuliginosus* (left lower) and *Miniopterus magnater* (upper and right lower). Photographs by Keping Sun and Tinglei Jiang.

Bent-winged bats *Miniopterus schreibersii* were previously considered to be widely distributed across the Old World ranging from Europe through to the Pacific (Koopman 1994; Wilson and Reeder 2005). The complex is now known to comprise several species (Appleton et al. 2004; Tian et al. 2004; Furman et al. 2009, 2010). In China, two bent-winged bat species, *Miniopterus fuliginosus* Hodgson, 1835 and *Miniopterus magnater* Sanborn, 1931 (Fig.1), were formerly confused with *M. schreibersii* (Hendrichsen et al. 2001; Appleton et al. 2004; Tian et al. 2004). Tian et al. (2004) argued that *M. schreibersii* from Guangxi and Hainan in China should be considered as *M. fuliginosus* based on intraspecific mtDNA divergence levels (Tian et al. 2004), which supported Maeda's recognition of Asian *Miniopterus schreibersii* as a distinct species, *M. fuliginosus* (Maeda 1982). Furthermore, Maeda regarded the bent-winged bats from Hainan Island to differ from individuals on the Chinese mainland (Maeda 1982). Those specimens originally labeled as *M. schreibersii *in South-East Asia may represent *M. magnater* (Hendrichsen et al. 2001). *Miniopterus magnater* has been recorded in southern China, including Hainan, Guangdong, Hongkong, and Fujian (Smith and Xie 2008) and shares similar morphological characteristics with *M. fuliginosus* in China (Maeda 1982; Smith and Xie 2008). Most of their morphological characters overlap, and the two species differ only in skull size, with *M. magnater* being slightly larger and wider than that of *M. fuliginosus*. Confusion of morphological characteristics between *M. fuliginosus* and *M. magnater* makes the mapping of the distribution extremely difficult in China, which suggest that bent-winged bat species distribution limits and characters need further research and evaluation.

Molecular data could play a major role in a re-examination of the taxonomics, phylogeny, and lineage divergences of the bent-winged bats. More recently, cytochrome c oxidase subunit I (COI) has been selected as the DNA bar codes for members of the animal kingdom (Hebert et al. 2003; Marshall 2005; Kerr et al. 2009) and has established a standardized approach to help field researchers in identifying species accurate (Borisenko et al. 2008). And COI bar codes are proved as an effective tool for both differentiating and identifying species of bats (Borisenko et al. 2008; Francis et al. 2010). Francis et al. (2010) have sequenced the COI bar codes of *M. fuliginosus* and *M. magnater* (Francis et al. 2010), which are available for clarifying the taxonomy and distribution range of the two species in China.

Glaciation and resultant geographic isolation might be considered as major mechanisms underlying the genetic differentiation of these two closely related bat species, *M. fuliginosus* and *M. magnater*. As a particular region, China encompasses both the Palearctic and Oriental biogeographic regions and occupies several climatic zones. Due to the influence of Pleistocene glacial cycles, many mammals, including bats, show distinct genetic differentiation among populations from different geographic regions and climatic zones (Ramos Pereira et al. 2009), implying the past existence of several glacial refugia (Huang et al. 2007) including eastern and southwestern regions in China (Yan et al. 2007).

In this study, to clarify the taxonomy, distributions, and genetic differentiation of Chinese bent-winged bats, *M. fuliginosus* and *M. magnater*, we collected 125 samples across the entire range of the two species in China as well as one site in Vietnam. Firstly, we used DNA barcoding, COI, to identify the taxonomy of *M. fuliginosus* and *M. magnater* and determine their distribution ranges. Secondly, we used sequences of the mitochondrial hypervariable control region (D-loop) and NADH dehydrogenase subunit 2 (ND2) to investigate their most recent common ancestor and divergent time. Thirdly, we inferred whether climatic oscillations in Pleistocene have affected the current distribution of bent-winged bats. Lastly, we investigated the difference of morphological characters of *M. fuliginosus* and *M. magnater*.

## Materials and Methods

### Sampling

**Figure 2:**
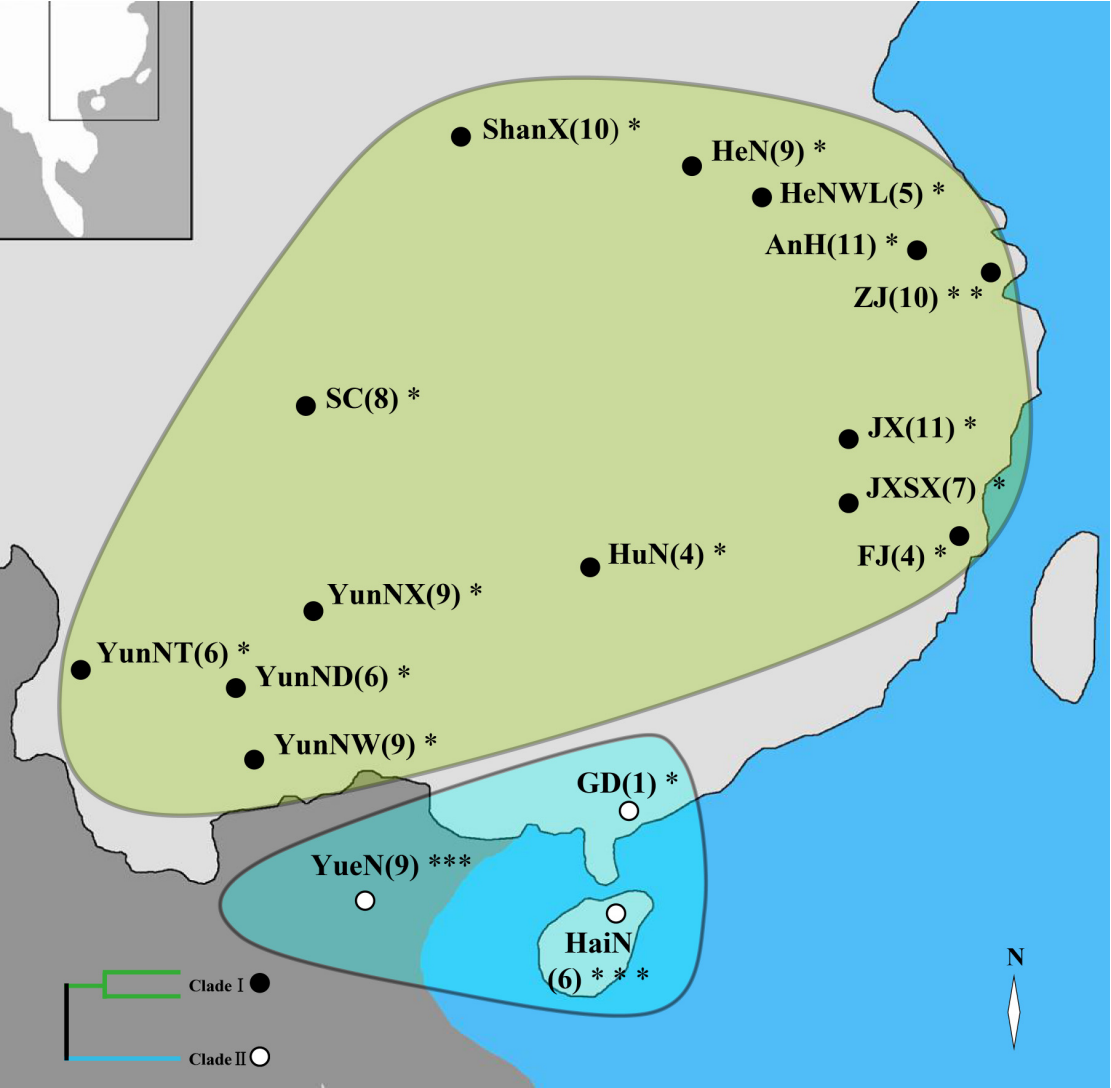
Sampling locations in this study. Map showing *Miniopterus fuliginosus* and *Miniopterus magnater* sampling sites. The different colors surrounded by solid lines correspond to the two species (*Miniopterus fuliginosus*: green; *Miniopterus magnater*: blue). Populations from which individuals were sampled for the ND2 and CO1 gene analysis are indicated by an asterisk. Numbers of sampled individuals are given in parentheses.

To encompass their entire range in China, we collected samples and morphological data of *M. fuliginosus* and *M. magnater* in China and Vietnam from 2007 to 2010. The sampling range extended across Palearctic and Oriental regions (Fig.2). After capturing the bats in a mist net, we measured their morphological characters with digital calipers (0.01 mm) and collected wing membranes using 3-mm biopsy punchers (Worthington and Barratt 1996). The samples were preserved in absolute ethanol and stored at −20°C. The bats were released in situ. Any bats that died unexpectedly were preserved in absolute ethanol and transported to the laboratory for skull preparation and measurement. Samples were stored at the School of Environment of Northeast Normal University, Changchun, China. All field studies followed the regulations of Wildlife Conservation of the People's Republic of China (Chairman Decree [2004] No. 24) and were approved by National Animal Research Authority in Northeast Normal University, China (approval number: NENU-20080416).

### DNA extraction, PCR amplification, and DNA sequencing

Total genomic DNA of 125 individuals from 17 sampling sites was extracted using a UNIQ-10 Column animal genomic DNA isolation kit (Sangon, Shanghai, China). To analyze the genetic differentiation of bent-winged bats, we amplified the mitochondrial D-loop region for all the 125 individuals from 17 sampling sites using the two universal primers P and E as described in Wilkinson and Chapman (1991). PCR amplifications were performed in 25 *μ*L volumes containing 2.5 *μ*L of 10 × PCR buffer, 2 *μ*L dNTP mixture (10 mmol/L), 1 *μ*L of each primer (10 *μ*mol/L), 1 *μ*L template DNA, and 0.5 *μ*L *Taq* polymerase (5 units/*μ*L). Samples were subjected to an initial denaturation step of 95°C for 5 min, followed by 40 cycles of denaturation at 95°C for 1 min, annealing at 55°C for 90 sec, and extension at 72°C for 2 min, followed by a final extension step of 72°C for 7 min. All haplotype sequences were deposited in GenBank under Accession Numbers KM230117–KM230241.

**Figure 3:**
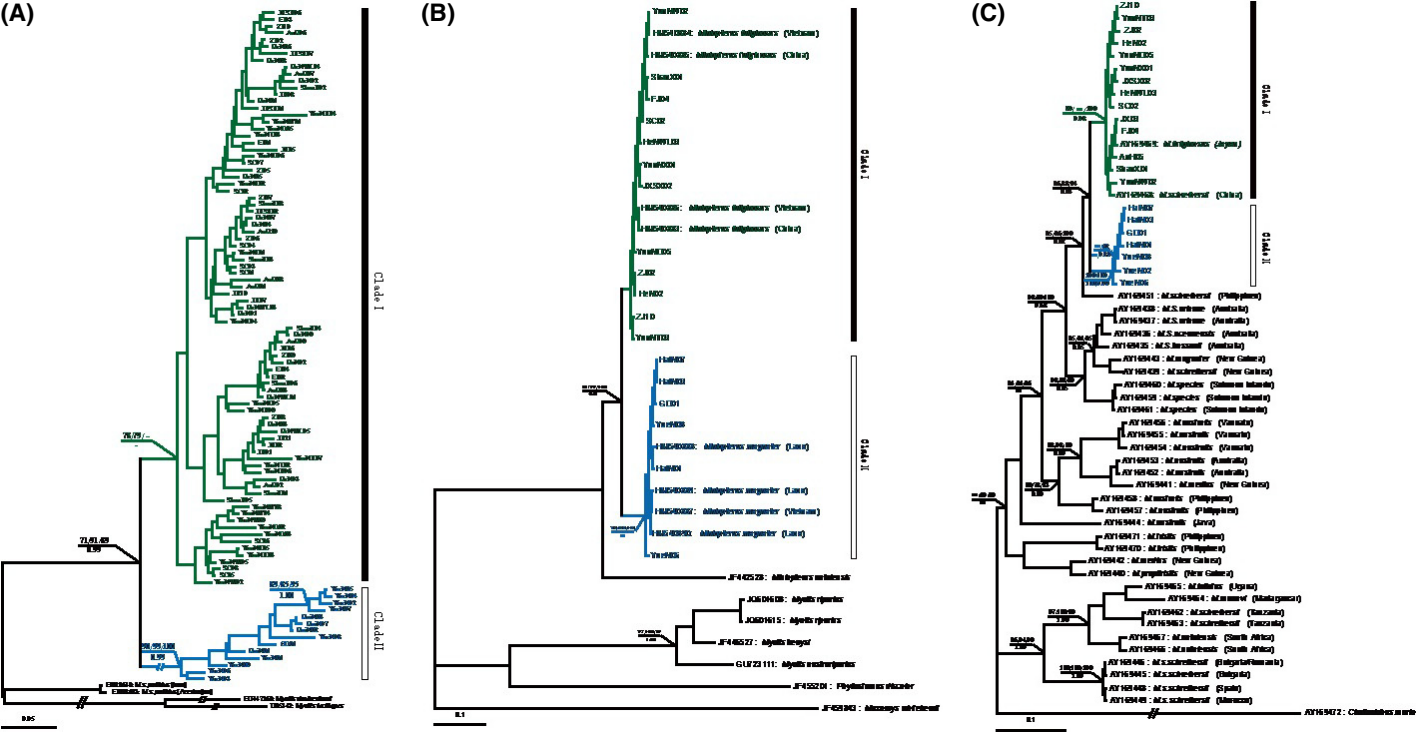
Phylogenetic trees recovered from maximum likelihood analysis of D-loop (A), CO1 (B) and ND2 (C) sequences. Bootstrap support and posterior probabilities are shown only for nodes highly supported by at least two phylogenetic reconstruction methods (i.e., ≥60% maximum likelihood, maximum parsimony or neighbor-joining bootstrap support or ≥0.60 Bayesian posterior probability). Sequences obtained from GenBank are indicated by their accession numbers and species names. Different clades are represented by different colors: green for *Miniopterus fuliginosus* and blue for *Miniopterus magnater*.

Based on mtDNA D-loop trees of bent-winged bats, two major monophyletic lineages were identified, corresponding to Chinese mainland and tropic region, respectively (Figs2 and 3A). To confirm the taxonomy of those bent-winged bats, we used primers (VF1_t1 and VR1_t1) and methods described in Borisenko et al. (2008) and Francis et al. (2010) to amplify the 657 bp segment of COI bar codes of 22 individuals, which were randomly selected from each sampling sites of Chinese mainland and tropic region (Fig.2). The resulting sequences (KM575709–KM575714, KM575717–KM575722, KP247536–KP247545) were compared with COI sequences of *M. fuliginosus* and *M. magnater* from other researches (HM540883–HM540890).

In order to analyze and compare with those ND2 sequences of bent-winged bats from Appleton et al. (2004), we also used primers (L5216F and H6313R) (Appleton et al. 2004) to amplify the entire 1037 bp ND2 gene of the same 22 individuals as COI bar codes (Fig.2). The resulting sequences (KM246394, KM246397–KM246400, KM246403–KM246407, KP247524–KP247535) were combined with ND2 sequences of other relevant taxa (AY169435–AY169472) for subsequent analysis.

All samples were sequenced by Sangon in Shanghai, China. The sequences were edited and aligned using Clustal × 1.8 (Thompson et al. 1997) followed by manual adjustments.

### Mitochondrial DNA analysis

Genetic diversity parameters such as haplotype diversity (*h*), nucleotide diversity (*π*), and the number of polymorphic and phylogenetically informative sites were calculated in DnaSP 5.10 (Rozas et al. 2003). Uncorrected genetic distances between and within different species were calculated using MEGA6 (Tamura et al. 2013).

For phylogenetic tree reconstruction, COI sequences of *Miniopterus natalensis* (JF442528), *Myotis austroriparius* (GU72311), *Myotis keaysi* (JF446527), *Phyllostomus discolor* (JF455201), *Maxomys whiteheadi* (JF459843), two *Myotis riparius* (JQ601608, JQ601615), D-loop sequences of *Myotis daubentonii* (EU447269), *Myotis lucifugus* (U95342), two *M. schreibersii pallidus* (FJ028633, FJ028640), and the ND2 sequence of *Chalinolobus morio* (AY169472) were used as out-groups, respectively. Optimal models of nucleotide substitution were determined using the Akaike Information Criterion (AIC) in jModelTest 0.1.1. The best models selected according to the AIC were TPM2uf + G for COI bar code, TPM1uf + I + Γ for the D-loop region, and TIM2 + I + G for the ND2 fragment. Phylogenetic analyses included maximum likelihood (ML) performed in PHYML (Guindon et al. 2005), maximum parsimony (MP) and neighbor-joining (NJ) in PAUP* 4.0 (Swofford 2001), and Bayesian inference (BI) in MrBayes 3.1.2. For the Bayesian analyses, two independent parallel runs of four incrementally heated Metropolis-coupled Monte Carlo Markov chains (MCMCs) were conducted, with trees sampled every 100 generations for 10,000,000 generations. The analyses were deemed to have converged when the average standard deviation of split frequencies fell below 0.01. The first 25% of the generations were discarded as “burn-in”. Statistical support for branching patterns under MP, NJ, and ML was estimated by 1000 bootstrap replications.

The time of the most recent common ancestor (TMRCA) of the two species based on D-loop and ND2 regions was assessed using BEAST 1.6.2 (Drummond and Rambaut 2007). Because the divergence rate for the D-loop of genus *Miniopterus* is unknown, we used a divergence rate of 20% per million years as applied in *Nyctalus* bats (Petit et al. 1999). For the mutation rate for ND2, we used 1.2 and 1.8% per million years as described for *Rhinolophus ferrumequinum* (Flanders et al. 2009). The best substitution models, estimated using jModeltest 0.1.1, were TPM1uf + I + Γ for the D-loop and TIM2 + I + G for ND2. Because TPM and TIM models of sequence evolution are not implemented in BEAST, we used the most similar model available. The prior parameters were determined in preliminary studies. Finally, we performed runs of 30,000,000 generations, each with a burn-in of the first 10% generations, with sampling every 1,000 steps. The results were then visualized in TRACER 1.5 (Drummond and Rambaut 2007), which was also used to examine the effective sample size (ESS) of each parameter for all ESSs >1000.

Neutrality tests and mismatch distribution analyses based on the D-loop sequences were used to infer population demographic events. For two mitochondrial lineages, the population demographic events of *M. fuliginosus* were analyzed, but *M. magnater* was not analyzed due to small sample size (*n* = 16). Fu's Fs (Fu 1997) and Fu and Li's F* and D* (Fu and Li 1993) were calculated in DnaSP 4.0. Mismatch distributions were calculated using 1,000 bootstrap replications in Arlequin. We used goodness-of-fit tests based on the sum of squared deviations (SSD) (Schneider and Laurent 1999) and raggedness index (Rogers and Harpending 1992) to test the significance of the fit of the distribution. When an expansion model could not be rejected, we estimated the time of expansion (*t*) from *τ *= 2*ut*, where *τ* is calculated as the time to expansion in mutational units, and *u* is the mutation rate per generation for the whole sequence. The values of *u* are equal to *μgk*, where *μ* is the mutation rate per nucleotide (see above) and *k* is the sequence length. The generation time (*g*) was estimated to be 2 years (Xu et al. 2010).

### Morphology analysis

To evaluate possible differentiations of the main morphological characters between *M. fuliginosus* and *M. magnater*, we analyzed forearm length (FA) for 97 samples obtained after 2007 and analyzed two major cranial parameters, maxillary third molar (M^3^) width and greatest length of the skull (GLS) for 19 unexpected dead bodies.

We used multidimensional scaling (MDS) to arrange FA, M^3^ width and GLS, between specimen pairs in a two-dimensional space. From the MDS plots, the FA, M^3^ width and GLS of all specimens clustered into two distinct groups with little overlap, which was consistent with the classification of three mtDNA marker. Analysis of variance (ANOVA) was therefore used to test differences between the two species. The FA of 97 individuals was measured from 13 populations (AnH, HeN, ZJ, JX, YunND, ShanX, HeNWL, SC, JXSX, FJ, YueN, GD, and HaiN). The two cranial parameters were measured in 5 individuals from Hainan and 14 individuals from the Chinese mainland (AnH, YunNX, YunNW, HuN, JX, and ZJ).

## Results

### Phylogeny and genetic divergence

Phylogenetic reconstructions from neighbor-joining (NJ), maximum parsimony (MP), maximum likelihood (ML), and Bayesian inference (BI) analyses produced highly concordant trees based on mitochondrial D-loop gene (Fig.3A). All individuals from China and Vietnam formed a monophyletic lineage with high bootstrap and posterior probability support (71% [ML], 91% [MP], 89% [NJ], and 0.99 [BI]). Within this monophyletic group, two major monophyletic lineages were identified, designated as Clade I from the Chinese mainland (ShanX, HeN, HenWL, AnH, ZJ, JX, JXSX, FJ, HuN, SC, YunNX, YunNT, YunND, and YunNW) and Clade II from tropical regions (Hainan, Guangdong, and Vietnam) (Figs2 and 3A).

Phylogenetic reconstructions from NJ, MP, ML, and BI analyses also produced highly concordant trees based on COI bar codes (Fig.3B). The individuals randomly selected from each population of Chinese mainland (Clade I) clustered together with *M. fuliginosus*, and the individuals from tropical regions (Clade II) clustered together with *M. magnater* (Fig.3B). The two lineages diverged by 7% based on uncorrected genetic distances, while intralineage divergences ranged from 0.1% to 1.4% for *M. fuliginosus*, and 0.1% to 1.8% for *M. magnater*. These results indicated that our samples from Chinese mainland was *M. fuliginosus*, and those from tropical regions was *M. magnater*.

The topology of ML, MP, and NJ trees based on mitochondrial ND2 was similar to those reported by Appleton et al. (2004). Similar to the D-loop trees and COI trees, all individuals clustered into two lineages, *M. fuliginosus* and *M. magnater* (Fig.3C). *M. fuliginosus* from the Chinese mainland and Japan (AY169469) formed a sister species with *M. magnater* from tropical regions (Fig.3C), but *M. magnater* from New Guinea was located in another clade (Fig.3C). The average genetic distance between *M. fuliginosus* (Clade I) and *M. magnater* (Clade II) was 5.3%. However, *M. magnater* from New Guinea was relatively genetically distant to Clade I (9.7%) and Clade II (10.3%) (Fig.3C).

### Genetic diversity and estimation of divergence and expansion time

Amplification of the D-loop from 125 individuals yielded 98 haplotypes with 98 polymorphic sites and 81 phylogenetically informative sites. The total haplotype diversity (*h*) was 0.99 (SD = 0.002), and the overall nucleotide diversity (*π*) was 0.07 (SD = 0.003). Haplotype and nucleotide diversities of each population are given in Table1. Haplotype (*h *=* *0.99, SD = 0.003) and nucleotide (*π *= 0.05, SD = 0.001) diversities of *M. fuliginosus* were higher than those of *M. magnater* (*h *=* *0.98, SD = 0.028; *π *= 0.04, SD = 0.003), which might be due to smaller sample sizes of *M. magnater* than *M. fuliginosus*.

**Table 1:** Genetic diversity parameters of sampled *Miniopterus fuliginosus* and *Miniopterus magnater* individuals. Species (Sp.), number of sampled individuals (*N*), haplotypes (*A*), and mean values of haplotype diversity (*H*_*d*_), nucleotide diversity (*π*) are shown in the table

Sp.	Population	*N*	D-loop		
			*A*	*H*_d_ ± SD	*π *± SD
*Miniopterus fuliginosus*	AnH	11	8	0.93 ± 0.066	0.05 ± 0.005
	HeN	9	9	1.00 ± 0.052	0.05 ± 0.007
	ZJ	10	10	1.00 ± 0.045	0.05 ± 0.005
	FJ	4	4	1.00 ± 0.177	0.05 ± 0.013
	YunNX	9	9	1.00 ± 0.052	0.06 ± 0.005
	YunNW	9	6	0.89 ± 0.091	0.03 ± 0.004
	JX	11	11	1.00 ± 0.039	0.05 ± 0.004
	YunND	6	5	0.93 ± 0.122	0.05 ± 0.008
	ShanX	10	9	0.98 ± 0.054	0.05 ± 0.007
	HuN	3	3	1.00 ± 0.272	0.07 ± 0.018
	YunNT	6	6	1.00 ± 0.096	0.05 ± 0.006
	HeNWL	5	5	1.00 ± 0.126	0.06 ± 0.009
	SC	8	8	1.00 ± 0.063	0.03 ± 0.006
	JXSX	7	7	1.00 ± 0.176	0.05 ± 0.006
*Miniopterus magnater*	GD	1	1	–	–
	HaiN	7	6	0.95 ± 0.096	0.05 ± 0.020
	YueN	9	9	1.00 ± 0.052	0.05 ± 0.005
	*Miniopterus fuliginosus*	109	84	0.99 ± 0.003	0.05 ± 0.001
	*Miniopterus magnater*	16	14	0.98 ± 0.028	0.04 ± 0.003
	Total	125	98	0.99 ± 0.002	0.07 ± 0.003

Based on D-loop, BEAST inferred the TMRCA for the two species of 2.26 Ma (95% highest probability density [HPD] = 1.23–3.48 Ma). Using a mutation rate of 1.2% and 1.8% per million years of ND2, their TMRCA was 2.37 Ma (95% HPD = 1.80–2.99 Ma) and 1.69 Ma (95% HPD = 1.25–2.15 Ma), respectively.

TMRCA for each species was calculated using each gene. Based on D-loop, TMRCA for *M. fuliginosus* was 0.77 Ma (95% HPD = 0.51–1.05 Ma) and for *M. magnater* was 0.58 Ma (95% HPD = 0.35–0.78 Ma). Using a mutation rate of 1.2% per million years of ND2, the TMRCAs for *M. fuliginosus* and *M. magnater* were 0.72 Ma (95% HPD = 0.53–0.96 Ma) and 0.59 Ma (95% HPD = 0.37–0.83 Ma). Using a mutation rate of 1.8% per million years, for *M. fuliginosus*, the estimated TMRCA was 0.50 Ma (95% HPD = 0.36–0.67 Ma), with a value of 0.42 Ma (95% HPD = 0.25–0.59 Ma) calculated for *M. magnater*.

In neutrality tests of *M. fuliginosus*, Fu and Li's F* and D* were not significant, whereas Fu's Fs was significant; these results suggest a population expansion model for its demographic history. At the same time, Harpending's raggedness index was small (*r *=* *0.002, *P *=* *0.97), indicating a rapid population expansion. The inferred expansion time was 0.14 Ma (0.09–0.19 Ma).

### Morphological divergence

**Figure 4:**
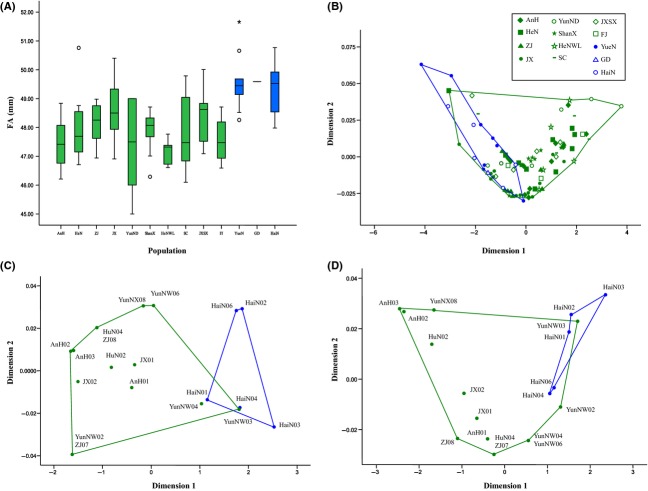
Variation in morphological data among populations in mtDNA clades. (A) Forearm length (FA) variation. Populations are arranged along the *x*-axis. For each box plot, the box represents the 0.25 quantile, median and 0.75 quantile. On either side of the box, the whiskers extend to the minimum and maximum values; (B) multidimensional scaling plot between pairs of specimens for FA; (C) multidimensional scaling plot between pairs of specimens for GLS; (D) multidimensional scaling plot between pairs of specimens for width across the maxillary third molar. The colors on the plot correspond to the two lineages in Fig.3.

We set up a two-dimensional MDS model to discriminate the difference of FA, GLS, and M^3^ width variation between *M. fuliginosus* and *M. magnater*. The first two dimensions extracted from the MDS model described nearly all the variation in FA, GLS, and M^3^ width between the two lineages (*r*^2^ = 0.99 for FA; *r*^2^ = 0.99 for GLS; *r*^2^ = 0.99 for M^3^ width) (Fig.4), which revealed two distinct geographic groups, *M. fuliginosus* and *M. magnater*, similar to the phylogenetic clusters. The mean values of FA length (47.92 ± 1.06 mm; *n *=* *81), GLS (16.09 ± 0.36 mm; *n *=* *14), and M^3^ width (6.71 ± 0.25 mm; *n *=* *14) in *M. fuliginosus* were significantly slightly smaller than in *M. magnater* (FA: 49.50 ± 0.97 mm, *n *=* *16; GLS: 16.91 ± 0.16 mm, *n *=* *5; M^3^ width: 7.12 ± 0.10 mm, *n *=* *5) (ANOVA, all *P *<* *0.01), even with little morphological overlap between the two species (Fig.4).

## Discussion

### Taxonomy of bent-winged bats

Our analyses indicate that DNA bar codes are an effective tool for differentiating and identifying species of bent-winged bats in China and Vietnam. Two divergent lineages (Fig.3B) and extremely low COI genetic divergences (< 2%) between our samples and *M. fuliginosus* HM540883–HM540886) or *M. magnater* (HM540887–HM540890) suggested that the bent-winged bats from Clade I are *M. fuliginosus*, and those from Clade II are *M. magnater*. This result is consistent with other previously studies on bent-winged bats’ taxonomy (Appleton et al. 2004; Tian et al. 2004), suggesting two bent-winged bat species exist in China. However, *M. magnater* from New Guinea was relatively genetically distant to *M. fuliginosus* (9.7%) and *M. magnater* (10.3%) from China and Vietnam based on ND2 gene (Fig.3C), which also suggested that further investigation is required in New Guinea to determine the correct name of bent-winged bats (Appleton et al. 2004).

### Potential mechanisms of spatial distribution

In this study, these two sister bent-winged bat species correspond to different spatial distributions, the Chinese mainland for *M. fuliginosus* and tropical regions for *M. magnater* (Figs2 and 3). Several mechanisms may be hypothesized to explain the current distribution pattern of *M. fuliginosus* and *M. magnater*, including palaeoclimatic changes (Jablonski and Whitfort 1999), climate differences (Miller-Butterworth et al. 2003; Bilgin et al. 2008), ecological attributes (Lin et al. 2014), and geographic isolation (Smissen et al. 2013).

A large body of evidence indicates that Pleistocene glacial cycles may have influenced differentiation, expansion, and genetic structure of different species (Taberlet et al. 1998; Hewitt 2000). In our study, the most recent common ancestor of *M. fuliginosus* and *M. magnater* was dated back to 2.26 Ma on the basis of the D-loop data and to 2.37–1.69 Ma according to the ND2 data. During this time period, China had just concluded an early stage of Quaternary glaciation (2.50 Ma) and was experiencing major climatic oscillations with a dominant 0.1-million-year cycle (Ruddiman et al. 1988; Liu et al. 2001). Climatic changes and temperature decline may have forced their ancestor to migrate southward and evolve in different refugia. Our study results indicate that the first divergence between these two species occurred either 2.26–0.58 Ma (D-loop data) or 2.37–0.42 Ma (ND2 data). This time frame extends across several glacial–interglacial stages, such as the Poyang glacial stage (1.8 Ma), the Dagu glacial stage (1.1 Ma) (Jing and Liu 1999), stages I (0.71–0.59 Ma) and II (0.52–0.46 Ma) of the third glaciation (counting backwards). Multiple glacial events and different climates in isolated refugia are possible explanations for the genetic differentiation of *M. fuliginosus* and *M. magnater* in China and Vietnam. Unfortunately, on the basis of our results, it is not possible to infer the past positions of glacial refugia for *M. fuliginosus* and *M. magnater* in China and Vietnam.

Pleistocene climate changes also affected population expansion events. Population expansion tests indicated that population expansion for *M. fuliginosus* occurred around 0.14 Ma, corresponding to the early stage (0.07–0.15 Ma) of the last interglacial period when the rising temperatures promoted population growth. Several subsequent glacial–interglacial cycles may have led to various periods of isolation and contraction of *M. fuliginosus* in China, consistent with its high genetic diversity (Table1).

With respect to each species’ geographic distribution, the range of *M. fuliginosus* corresponds to subtropical and temperate zones with a relatively cold, dry climate, whereas *M. magnater* has a distribution restricted to tropical coastal areas that are milder and more humid. Differing precipitation and temperature regimes in the two regions might influence the distribution of vegetation as well as insect density and composition. In our study, the two species experiencing different climatic conditions may have originally occupied different habitats and climate zones because of local ecological adaptation, and then diverged after Quaternary glaciation. This type of association between different biomes and climatic conditions has been found in two other congeneric species, *M. natalensis* in South Africa (Miller-Butterworth et al. 2003) and *M. schreibersii pallidus* in southeastern Turkey (Bilgin et al. 2008), *Miniopterus manavi* in Madagascar and Comoros (Goodman et al. 2009), *Miniopterus fraterculus* (Goodman et al. 2007), and other *Miniopterus* species (Christidis et al. 2014) in Madagascar, indicating that environmental conditions can influence the distribution of bats.

The ecological attributes of *M. fuliginosus* and *M. magnater* have also played an important role in modulating contemporary geographic distribution pattern. These two species have high wing loading and a high wing aspect ratio. Normally, species with this wing morphology have long-range migratory and dispersal abilities, but these two species typically roosts in caves and feeds in forests (Han et al. 2008; Hu et al. 2011). The availability of cave habitat and food resources may have caused the two species to become isolated in refugia during past ice ages. However, potential biogeographic barriers constraining other bat species (Flanders et al. 2011), such as the Qinling Mountains and the Huaihe River separating Palearctic and Oriental regions (Xu et al. 2007), did not constitute effective barriers for *M. fuliginosus* and *M. magnater* in China.

### Morphology difference

Although *M. fuliginosus* and *M. magnater* can be identified based on genetic data, it is difficult to distinguish them in the field from living individuals because of their overlapping morphological characters, which is very common in Genus *Miniopterus* bat species, such as *M. schreibersii* and *M. maghrebensis,* or *M. pallidus* (Furman et al. 2009; Bilgin et al. 2012, Puechmaille et al. 2014). In this study, we found that the FA, M^3^ width, and GLS were significantly different between *M. fuliginosus* and *M. magnater*, with individuals from *M. magnater* significantly larger than those of *M. fuliginosus*. This result suggested that these three parameters could be used to identify taxonomy, but still need to combine the DNA sequence because of partial overlapping. However, the width of M^3^ across all individuals in our study was less than 7.3 mm (*M. magnater* is 7.03–7.29 mm, *M. fuliginosus* is 6.33–7.16 mm), whereas the width of M^3^ in *M. magnater* was reported greater than 7.4 mm by literature (Smith and Xie 2008).

## Conclusions

*M. fuliginosus* and *M. magnater* as sister species exist in China, extending across the Tropic of Cancer display a north–south distribution pattern corresponding to subtropical and temperate zones and tropical coastal areas, respectively. The TMRCA of *M. fuliginosus* and *M. magnater* could date back to the early Quaternary glacial period, with subsequent evolution occurring in different refugia. Both climate changes and their ecological attributes might have also played important roles in modulating geographic distribution pattern. Three main morphological characters, FA, M^3^ width, and GLS were significantly different between *M. fuliginosus* and *M. magnater,* and the latter was significantly larger than the former.

## References

[b1] Appleton BR, Mckenzie JA, Christidis L (2004). Molecular systematics and biogeography of the bent-wing bat complex *Miniopterus schreibersii* (Kuhl, 1817) (Chiroptera: Vespertilionidae). Mol. Phylogenet. Evol.

[b2] Bilgin R, Karatas A, Coraman E, Disotell T, Morales JC (2008). Regionally and climatically restricted patterns of distribution of genetic diversity in a migratory bat species, *Miniopterus schreibersii* (Chiroptera: Vespertilionidae). BMC Evol. Biol.

[b101] Bilgin R, Gürün K, Maraci Ö, Furman A, Hulva P, Çoraman E, Lu?an RK, Bartoni?ka T, Horá?ek I (2012). Syntopic occurrence in Turkey supports separate species status for *Miniopterus schreibersii schreibersii and M. schreibersii pallidus* (Mammalia: Chiroptera). Acta Chiropterologica.

[b3] Borisenko AV, Lim BK, Ivanova NV, Hanner RH, Hebert PD (2008). DNA barcoding in surveys of small mammal communities: a field study in Suriname. Mol. Ecol. Resour.

[b4] Christidis L, Goodman SM, Naughton K, Appleton B (2014). Insights into the evolution of a cryptic radiation of bats: dispersal and ecological radiation of Malagasy *Miniopterus* (Chiroptera: Miniopteridae). PLoS ONE.

[b5] Drummond AJ, Rambaut A (2007). Beast: Bayesian evolutionary analysis by sampling trees. BMC Evol. Biol.

[b6] Flanders J, Jones G, Benda P, Dietz C, Zhang S, Li G (2009). Phylogeography of the greater horseshoe bat, *Rhinolophus ferrumequinum*: contrasting results from mitochondrial and microsatellite data. Mol. Ecol.

[b7] Flanders J, Wei L, Rossiter SJ, Zhang S (2011). Identifying the effects of the Pleistocene on the greater horseshoe bat, *Rhinolophus ferrumequinum*, in East Asia using ecological niche modelling and phylogenetic analyses. J. Biogeogr.

[b8] Francis CM, Borisenko AV, Ivanova NV, Eger JL, Lim BK, Guillén-Servent A (2010). The role of DNA barcodes in understanding and conservation of mammal diversity in southeast Asia. PLoS ONE.

[b9] Fu YX (1997). Statistical tests of neutrality of mutations against population growth, hitchhiking and background selection. Genetics.

[b10] Fu YX, Li WH (1993). Statistical tests of neutrality of mutations. Genetics.

[b11] Furman A, Coraman E, Bilgin R, Karatas A (2009). Molecular ecology and phylogeography of the bent-wing bat complex (*Miniopterus schreibersii*) (Chiroptera: Vespertilionidae) in Asia Minor and adjacent regions. Zoolog. Scr.

[b12] Furman A, Postawa T, Oztunc T, Coraman E (2010). Cryptic diversity of the bent-wing bat, *Miniopterus schreibersii* (Chiroptera: Vespertilionidae), in Asia Minor. BMC Evol. Biol.

[b13] Goodman SM, Ryan KE, Maminirina CP, Fahr J, Christidis L, Appleton B (2007). Specific status of populations on madagascar referred to *Miniopterus fraterculus* (Chiroptera: Vespertilionidae), with description of a new species. J. Mammal.

[b14] Goodman SM, Maminirina CP, Weyeneth N, Bradman HM, Christidis L, Ruedi M (2009). The use of molecular and morphological characters to resolve the taxonomic identity of cryptic species: the case of *Miniopterus manavi* (Chiroptera: Miniopteridae) on Madagascar and the Comoros. Zoolog. Scr.

[b15] Guindon S, Lethiec F, Duroux P, Gascuel O (2005). Phyml online—a web server for fast maximum likelihood-based phylogenetic inference. Nucleic Acids Res.

[b16] Han BY, Hua PY, Gu XM, Miller-Butterworth CM, Zhang SY (2008). Isolation and characterization of microsatellite loci in the long-fingered bat *Miniopterus fuliginosus*. Mol. Ecol. Resour.

[b17] Hebert PDN, Cywinska A, Ball SL, Dewaard JR (2003). Biological identifications through DNA barcodes. Proc. R. Soc. Lond. B Biol. Sci.

[b18] Hendrichsen DK, Bates PJJ, Hayes BD, Walston JL (2001). Recent records of bats (Mammalia: Chiroptera) from Vietnam with six species new to the country. Myotis.

[b19] Hewitt G (2000). The genetic legacy of the Quaternary ice ages. Nature.

[b20] Hu KL, Wei L, Zhu TT, Wang XZ, Zhang LB (2011). Dietary composition, echolocation pulses and morphological measurements of the long-fingered bat *Miniopterus fuliginosus* (Chiroptera: Vespertilioninae). Zool. Res.

[b21] Huang Z, Liu N, Luo S, Long J (2007). Phylogeography of rusty-necklaced partridge (*Alectoris magna*) in northwestern China. Mol. Phylogenet. Evol.

[b23] Jablonski NG, Whitfort MJ (1999). Environmental change during the Quaternary in East Asia and its consequences for mammals. Rec. West. Aust. Mus. Suppl.

[b24] Jing CR, Liu HP (1999). On the glacial and interglacial stages in Quaternary of China. J. Cheng Du Univ. Tech.

[b25] Kerr KCR, Lijtmaer DA, Barreira AS, Hebert PDN, Tubaro PL (2009). Probing evolutionary patterns in neotropical birds through DNA barcodes. PLoS ONE.

[b26] Koopman KF (1994). Chiroptera: systematics handbook of zoology volume VIII mammalia.

[b27] Liu J, Ni Y, Chu G (2001). Main palaeoclimatic events in the Quaternary. Quat. Sci.

[b100] Lin AQ, Emerson B, Csorba G, Li LF, Jiang TL, Lu GJ, Thong VD, Soisook P, Sun KP, Feng J (2014). Phylogeography of *Hipposideros armiger* (Chiroptera: Hipposideridae) in the Oriental Region: the contribution of multiple Pleistocene glacial refugia and intrinsic factors to contemporary population genetic structure. Journal of Biogeography.

[b28] Maeda K (1982). Studies on the classification of *Miniopterus* in Eurasia, Australia and Melanesia. Honyurui Kagaku (Mammalian Science).

[b29] Marshall E (2005). Will DNA bar codes breathe life into classification?. Science.

[b30] Miller-Butterworth CM, Jacobs DS, Harley EH (2003). Strong population substructure is correlated with morphology and ecology in a migratory bat. Nature.

[b31] Petit E, Excoffier L, Mayer F (1999). No evidence of bottleneck in the postglacial recolonization of Europe by the noctule bat (*Nyctalus noctula*. Evolution.

[b33] Puechmaille SJ, Allegrini B, Benda P, Gürün K, Srámek J, Ibañez C (2014). A new species of the *Miniopterus schreibersii* species complex (Chiroptera: Miniopteridae) from the Maghreb Region, North Africa. Zootaxa.

[b34] Ramos Pereira MJO, Salgueiro P, Rodrigues L, Coelho MM, Palmeirim JM (2009). Population structure of a cave-dwelling bat, *Miniopterus schreibersii*: does it reflect history and social organization. J. Hered.

[b35] Rogers AR, Harpending H (1992). Population growth makes waves in the distribution of pairwise genetic differences. Mol. Biol. Evol.

[b37] Rozas J, Sánchez-DelBarrio JC, Messeguer X, Rozas R (2003). Dnasp, DNA polymorphism analyses by the coalescent and other methods. Bioinformatics.

[b38] Ruddiman WF, Raymo ME, Lamb HH, Andrews JT (1988). Northern hemisphere climate regimes during the past 3 Ma: possible tectonic connections [and discussion]. Philos. Trans. R Soc. B Biol. Sci.

[b39] Schneider S, Laurent E (1999). Estimation of past demographic parameters from the distribution of pairwise differences when the mutation rates vary among sites: application to human mitochondrial DNA. Genetics.

[b40] Smissen PJ, Melville J, Sumner J, Jessop TS, Ebach M (2013). Mountain barriers and river conduits: phylogeographical structure in a large, mobile lizard (Varanidae: *Varanus varius*) from eastern Australia. J. Biogeogr.

[b41] Smith AT, Xie Y (2008). A guide to the mammals of China.

[b42] Swofford DL (2001). Paup: phylogenetic analysis using parsimony (and other methods).

[b43] Taberlet P, Fumagalli L, Wust-Saucy AG, Cosson JF (1998). Comparative phylogeography and postglacial colonization routes in Europe. Mol. Ecol.

[b44] Tamura K, Stecher G, Peterson D, Filipski A, Kumar S (2013). Mega6: molecular evolutionary genetics analysis version 6.0. Mol. Biol. Evol.

[b45] Thompson JD, Gibson TJ, Plewniak F, Jeanmougin F, Higgins DG (1997). The Clustal_X windows interface: flexible strategies for multiple sequence alignment aided by quality analysis tools. Nucleic Acids Res.

[b46] Tian LX, Liang B, Maeda K, Metzner W, Zhang SY (2004). Molecular studies on the classification of *Miniopterus schreibersii* (Chiroptera:Vespertilionidae) inferred from mitochondrial cytochrome b sequences. Folia Zool.

[b47] Wilkinson GS, Chapman AM (1991). Length and sequence variation in evening bat D-loop mtDNA. Genetics.

[b48] Wilson DE, Reeder DM (2005). Mammal species of the world: a taxonomic and geographic reference.

[b49] Worthington WJ, Barratt E (1996). A non-lethal method of tissue sampling for genetic studies of chiropterans. Bat Res. News.

[b50] Xu MF, Gao JJ, Chen HW (2007). Genus *Amiota Loew* (Diptera: Drosophilidae) from the Qinling mountain system, central China. Entomol. Sci.

[b51] Xu LJ, He CF, Shen C, Jiang TL, Shi LM, Sun KP (2010). Phylogeography and population genetic structure of the great leaf-nosed bat (*Hipposideros armiger*) in China. J. Hered.

[b52] Yan H, Peng C, Hu C, Hao G (2007). Phylogeographic structure of *Primula obconica* (Primulaceae) inferred from chloroplast microsatellites (cpSSRs) markers. Acta Phytotax. Sin.

